# Leachability of Arsenic and Heavy Metals from Mine Tailings of Abandoned Metal Mines

**DOI:** 10.3390/ijerph6112865

**Published:** 2009-11-17

**Authors:** Mihee Lim, Gi-Chun Han, Ji-Whan Ahn, Kwang-Suk You, Hyung-Seok Kim

**Affiliations:** Korea Institute of Geoscience and Mineral Resources (KIGAM), 92 Gwahang-no, Yuseong-gu, Daejeon 305-350, Korea; E-Mails: limmh@paran.com (M.L.); hsue@dreamwiz.com (G-C.H.); youks@kigam.re.kr (K-S.Y.); hskim@kigam.re.kr (H-S.L.)

**Keywords:** mine tailings, abandoned mines, arsenic (As), heavy metals, sequential extraction, EPA method 6010, Toxicity Characteristic Leaching Procedure (TCLP)

## Abstract

Mine tailings from an abandoned metal mine in Korea contained high concentrations of arsenic (As) and heavy metals [e.g., As: 67,336, Fe: 137,180, Cu: 764, Pb: 3,572, and Zn: 12,420 (mg/kg)]. US EPA method 6010 was an effective method for analyzing total arsenic and heavy metals concentrations. Arsenic in the mine tailings showed a high residual fraction of 89% by a sequential extraction. In Toxicity Characteristic Leaching Procedure (TCLP) and Korean Standard Leaching Test (KSLT), leaching concentrations of arsenic and heavy metals were very low [e.g., As (mg/L): 0.4 for TCLP and 0.2 for KSLT; cf. As criteria (mg/L): 5.0 for TCLP and 1.5 for KSLT].

## 1. Introduction

Mining operations generally produce many types of mine wastes, including mine tailings, waste rock and slag. Mine tailings out of those, in particular, act as a main source of environmental contamination [1]. Arsenic (As) and heavy metals may be released from the mine wastes to the ground and surface water systems, as well as the geological environment due to their solubility and mobility [2].

Mine tailings often contain sulfide minerals such as pyrite (FeS_2_), arsenopyrite (FeAsS), galena (PbS), chalcopyrite (CuFeS_2_), and sphalerite ((Fe, Zn)S). Oxidation, dissolution, precipitation, adsorption, and desorption mainly occur in mine tailings exposed to the air. Oxidation of sulfide minerals results in contamination of the surrounding soil and groundwater by allowing release of As and heavy metals in sulfide-bearing minerals [3–5]. Furthermore, As and heavy metals from mine tailings may cause fatal diseases in humans through crops and water due to the characteristic easy accumulation in internal organs [6].

Most of the mines in Korea have been closed since the 1970s due to various changes in the structure of the domestic industry. In investigations on actual conditions of 219 abandoned metal mines until 2006 among 936 ones nationwide, it has been revealed that As and heavy metals (e.g., Cu, Pb and Cd) concentrations in mine wastes at 139 abandoned metal mines (about 63%) are higher than the current Korean soil contamination criteria [7]. The criteria include both warning and acting criteria. In particular, the acting criteria express a serious pollution state needing control action such as the suspension of land development, owing to concerns on human health and growth of animals and plants. On the other hand, the warning criteria are 40% values of the action criteria and its objective is to prevent more serious soil contamination than the current state.

**Figure 1.**
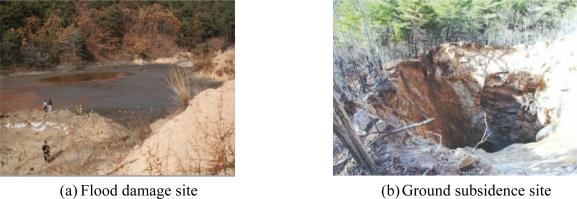
Kumho mine located in Bonghwa, Korea.

Based on the national investigation, among several abandoned mines it is well known that the wastes of the Kumho mine, located in Bonghwa, Kyoungbuk province, Korea, contain high concentrations of both As and heavy metals. The mine had generally produced Au, Ag, Cu, Pb, Zn, and Mn, and in particular produced about 140,000 tons of Mn until 1990s and 10,000–20,000 tons of Zn ore until early 2000s, but it was closed in 2001. A large amount of mine wastes including mine tailings, slag, and waste rock was also produced by the mining operation. Also, flood damages and ground subsidence have occurred at the mine several times because it was not equipped with any facility for the prevention of the damage (Figure 1). Therefore, the government recently started out to restore nearby water and soil environment of the mine. This abandoned mine, in this study, was selected for a sampling site to understand their characteristics and to estimate the application possibility of our technology, which will be explained below, to the ongoing restoration site.

In order to treat and prevent environmental pollution by mine wastes from abandoned metal mines, various treatment technologies such as soil washing [8], chemical reduction/oxidation [9], solidification/stabilization [10], bioremediation [11], *etc.* have been applied to contaminated sites. Our future research will be, in particular, focused on solidification/stabilization of heavy metals in the mine wastes and the soil by carbonation technology using Ca material and CO_2_ gas, which is our ultimate research goal [12–14]. For the future study, current investigation on contamination level and leaching potential of arsenic and heavy metals from the mine tailings would be a preliminary work for determining whether we can apply the carbonation to the specific mine tailings treatment.

In this study, three standard methods were used for determining total concentrations of As and heavy metals (Fe, Cu, Pb, Mn, Cr, Cd, and Zn) in mine tailings as follows: US EPA Standard Method (EPA method 6010), Korean Soil Environment Standard Test (KST for soil), and Korean Waste Standard Test (KST for waste). In addition, chemical combination characteristic of As in the mine tailings was analyzed by chemical extraction method of six steps. Leaching potential of As and heavy metals from the mine tailings was examined by both Toxicity Characteristic Leaching Procedure (TCLP; EPA method 1311) and Korean Standard Leaching Test (KSLT).

## 2. Experimental

A sampling of mine tailings was performed at the Kumho mine in April 2009. A small pile of the mine tailings was placed near a mine-head without any cover for the prevention of their loss. About 20 kg of the mine tailings were collected from the surface of the pile using a shovel. Mine tailings characteristics according to the pile depth was not considered in this study. The sample was put into plastic bags in the field. After transferring to our laboratory, the sample was sieved through a 5 mm-sieve, homogenized, and dried at 40 °C for two days. The sample was then stored in desiccators during the whole experimental period.

Several tests were performed to investigate the characteristics of the sample. All tests were repeated on two different days. Firstly, pH (520A, Orion) of the sample was measured after shaking at 100 rpm for an hour [wet sample: water = 1:10 (wt:wt)]. Water content, loss on ignition (LOI), and organic carbon content were also examined as methods that form part of the KST for waste [15]. Concentrations of anions in the mine tailings were analyzed using ion chromatography (IC; ICS 2000). The solution used in anion analysis was same as that in pH analysis. Chemical composition of the mine tailings was analyzed using an X-ray diffraction analyzer (XRD; X’pert, Phillips).

Total concentrations of As and heavy metals (Fe, Cu, Pb, Mn, Cr, Cd, and Zn) in the sample were determined. Based on results of previous researches indicating that concentrations of heavy metals can be different according to the testing methods used, we also applied three methods to our experiments: EPA method 6010, KST for soil, and KST for waste [15–17]. Both EPA method 6010 and KST for waste are methods involving digestion of soil, sediment, and sludge with HNO_3_, HCl, and H_2_O_2_ (no addition in KST for waste) on a hot plate, but the specific details of the two methods are somewhat different, as shown in Table 1. KST for soil is, on the other hand, a leaching method with 0.1 N HCl (for Cd, Cu, and Pb) or 1 N HCl (for As) at 100 rpm for an hour. In the three methods, final suspended solution was filtrated with 0.45 μm-membrane filter. Each filtrated solution was acidified (pH < 2) with two drops of 60% HNO_3_ and was analyzed using an atomic absorption spectrometer (AAS; AA-6800, Shimadzu).

A sequential extraction was also performed to operationally fractionate As in the solid materials to evaluate its potential effects [18]. A method developed by Herreweghe *et al.* [19] was applied in this study and it involves the following six steps: (1) easily soluble fraction by 1 M-NH_4_Cl 30 mL (2 hr shaking), (2) extractable fraction by 0.5 M-NH_4_F 30 mL (15 hr shaking), (3) extractable fraction by 0.1 M-NaOH 30 mL (17 hr shaking), (4) reducible fraction by 0.5 M-sodium citrate 30 mL and 1 M-NaHCO_3_ 2.5 mL while adding 0.5g-Na_2_S_2_O_4_·2H_2_O (15 min heating), (5) acid soluble fraction by 0.25 M-H_2_SO_4_ 30 mL (12 hr shaking), and 6) residual fraction by concentrated HCl 4 mL, HNO_3_ 2 mL and HF 2 mL (heating until half dry). In this test, 0.5 g of the mine tailings was placed in a 100 mL beaker. At each step, the suspension was filtrated with a 0.45 μm-membrane filter. The filtrate was used for arsenic analysis and the solid on the filter paper was used in the next step after washing with 20 mL of ultra-pure water. Then, the water used in washing was discharged.

Lastly, leaching concentrations of arsenic and heavy metals from the mine tailings were evaluated by both TCLP and KSLT [15,20]. The purpose of TCLP is to determine if a waste meets a hazardous waste code listed in 40CFR (Code of Federal Regulations) Part 26 under Resource Conservation and Recovery Act (RCRA), and that of KSLT is to determine if a waste is a hazardous matter specified over the regulation level of Korean waste management act or which landfill method is proper for a waste. The comparison between the two methods is shown in Table 2. In TCLP, fluid #1 was prepared by adding 5.7 mL of glacial acetic acid and 64.3 mL of 1 N NaOH to 500 mL of ultra-pure water and diluting total volume into 1 L with water, and fluid #2 was prepared by the same method as fluid #1 but without adding 1 N NaOH. To select a solution between #1 and #2, pH of the mine tailings was measured by shaking 5g of mine tailings with 96.5 mL for 5 min. Then, if pH of the solution is <5.0, fluid #1 is selected, otherwise following additional step is needed: the solution is acidified with 3.5 mL of 1 N HCl and heated at 50°C for 10 min. After cooling, if pH of the acidified solution is <5.0, fluid #1 is selected, otherwise fluid #2.

## 3. Results and Discussion

### 3.1. Physical and Chemical Characteristics of the Mine Tailings

The mine tailings showed dark-gray coloration. The sample was in a slightly wet state containing 9.6% water and its pH value was 7.5. The value of loss on ignition (LOI), which indicates the proportion of total organic matters, was 9.3% and organic carbon content among all organic matters was 3.9%. Concentrations of anions in the mine tailings analyzed using IC are shown in Table 3. Then, the concentrations of most anions (F^−^, Cl^−^, NO_2_^−^, NO_3_^−^, Br^−^, and PO_4_^2−^) were very low, whereas the concentration of SO_4_^2−^ (sulfate: 224 mg/L) was much higher than others. The high concentration of sulfate indicates that the oxidation of sulfide minerals had occurred in the mine tailings, so that sulfate had been produced. According to XRD analysis, the mine tailings mostly consist of quartz (SiO_2_), kaolinite (Al_2_Si_2_O_5_(OH)_4_), jarosite (KFe_3_(OH)_6_(SO_4_)_2_), pyrite (FeS_2_) and ferric hydroxide (Fe(OH)_3_).

### 3.2. Total Concentrations of As and Heavy Metals in the Mine Tailings

In order to measure total concentrations of As and heavy metals in the mine tailings, three standard USA and Korean methods were used: EPA method 6010, KST for soil, and KST for waste, as shown in Table 4 [15–17]. Total concentrations of As and heavy metals were significantly higher than Korean soil contamination criteria. This result indicates that the mine tailings were greatly contaminated and need appropriate treatments to prevent secondary contamination.

Total concentrations of As and heavy metals evaluated by EPA method 6010 and KST for waste were much higher than those evaluated by KST for soil. This result was consistent with that reported by Jung *et al.* [22]. In their study, extraction tests by 0.1N HCl solution (a similar method to KST for soil) and *aqua regia* (concentrated HCl and HNO_3_; a similar method to EPA method 6010 and KST for waste) were applied for determining total concentrations of heavy metals in mine wastes from several abandoned metalliferous mines in Korea. Then the concentrations of most heavy metals extracted by *aqua regia* were much higher than those by 0.1 N HCl, showing the similar tendency to our results. Another remarkable point observed in Table 4 is that, in particular, concentrations of As, Cu, Pb, and Cd obtained by EPA method 6010 are very similar to those by KST for waste (e.g., As: 67,336 mg/kg by EPA method 6010 and 66,155 mg/kg by KST for waste). However, As concentration extracted by KST for soil was only 5% of those by other methods.

These results are attributed to methodological differences between the three standard methods as shown in Table 1 in “Experimental” section. In other words, both EPA method 6010 and KST for waste are to digest all metals in a sample with very strong acids by heating under boiling point. Whereas KST for soil is to just extract metals by shaking at 30 °C with acidic solutions and is appropriate to the determination of only available (extractable) metal concentrations in a sample. Our suggestion for the analysis of metal concentration by the KST for soil is, therefore, that it should be either defined as a method to determine only available (extractable) metal concentrations in the soil, or modified as the other two methods to determine total metal concentration in the soil.

### 3.3. Chemical Distribution of As in the Mine Tailings by Sequential Extraction

**Figure 2.**
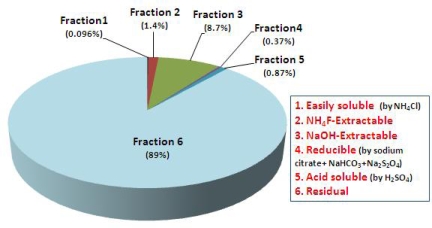
Chemical distribution of arsenic (As) in the mine tailings (Fraction 1 < Fraction 4 < Fraction 5 < Fraction 2 < Fraction 3 < Fraction 6).

**Figure 3.**
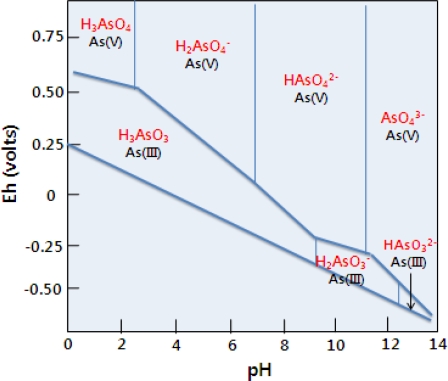
Speciation of arsenic (As) in water depending on pH (Eh: the oxidation/reduction potential (ORP) of the water) (modified from [23]).

Table 5 includes As contents, given in mg/L leachate, mg/Kg tailings, and % of total content, obtained from each step and As minerals (both name and formula) which may be extractable at each step for the mine tailings extracted in the six step sequence developed by Herrewghe *et al.* [19]. Figure 2 shows the proportion of each As fraction for total 100%. It is observed that most As in the mine tailings consists of the residual fraction (89%; 44,023 mg/kg), which is assumed to represent As hosted by silicate or sulfide minerals and extracted by heating with concentrated strong acids. NH_4_F-extractable fraction, As bound to aluminum (Al), was then 1.4% (716 mg/kg) and NaOH-extractable fraction, As bound to iron (Fe), was 8.7% (4,343 mg/kg), whereas the easily soluble fraction, weakly sorbed non-ionic As fraction, was only 0.096% (48 mg/kg). In this step, As_2_O_3_ (+III) and As_2_O_5_ (+V) in the mine tailings, which are hygroscopic, dissolve readily in the extractant; in particular they ionize into the forms of H_2_AsO_3_^−^ and HAsO_4_^2−^ at the neutral or weak alkaline pH, due to their ionization characteristic depending on pH as below equations 1–5[23]. Also, Figure 3 illustrates that speciation characteristic of As depending on pH is associated with its Eh values [23].

(1) $$\text{As}(\text{III}):{\text{H}}_{3}{\text{AsO}}_{3}\;\to \;{\text{H}}^{+}\;+\;{\text{H}}_{2}{\text{AsO}}_{3}^{-}\;\;\;\;\;\;\;\;\;\;\;\;\;\;\;\;\;\;\;\;\;\;\;\;\;\;\;\;\;\;\;\;\;\;\;\;\;\;\;\;\;{\text{pK}}_{\text{a}}\;=\;9.22$$

(2) $${\text{H}}_{2}{\text{AsO}}_{3}^{-}\;\to \;{\text{H}}^{+}\;+\;{\text{HAsO}}_{3}^{2-}\;\;\;\;\;\;\;\;\;\;\;\;\;\;\;\;\;\;\;\;\;\;\;\;\;\;\;\;\;\;\;\;\;\;\;\;\;\;\;\;\;{\text{pK}}_{\text{a}}\;=\;12.3$$

(3) $$\text{As}(\text{V}):\;\;{\text{H}}_{3}{\text{AsO}}_{4}\;\to \;{\text{H}}^{+}\;+\;{\text{H}}_{2}{\text{AsO}}_{4}^{-}\;\;\;\;\;\;\;\;\;\;\;\;\;\;\;\;\;\;\;\;\;\;\;\;\;\;\;\;\;\;\;\;\;\;\;\;\;\;\;\;\;{\text{pK}}_{\text{a}}\;=\;2.2$$

(4) $${\text{H}}_{2}{\text{AsO}}_{4}^{-}\;\to \;{\text{H}}^{+}\;+\;{\text{HAsO}}_{4}^{2-}\;\;\;\;\;\;\;\;\;\;\;\;\;\;\;\;\;\;\;\;\;\;\;\;\;\;\;\;\;\;\;\;\;\;\;\;\;\;\;\;\;{\text{pK}}_{\text{a}}\;=\;7.08$$

(5) $${\text{HAsO}}_{4}^{2-}\;\to \;{\text{H}}^{+}\;+\;{\text{AsO}}_{4}^{3-}\;\;\;\;\;\;\;\;\;\;\;\;\;\;\;\;\;\;\;\;\;\;\;\;\;\;\;\;\;\;\;\;\;\;\;\;\;\;\;\;\;{\text{pK}}_{\text{a}}\;=\;11.5$$

where, pK_a_ is the pH at which the dissociation of the reactant reaches 50%.

In terms of methodology of the As sequential extraction, results are representative of As only in a qualitative sense and may determine the amount of surface-bound As. It, however, does not indicate either the information on As species in relatively insoluble precipitates or the composition and geometry of adsorbed As complexes [18]. Based on the knowledge, these results shown in Table 5 should be considered as data for estimating the distribution of As fractions which can be extractable by the specified acidic and alkaline solutions.

On the other hand, it is observed that an order in the contents of the extractable fractions is NaOH (Step 3) ≫ NH_4_F (Step 2) > H_2_SO_4_ (Step 5) > NH_4_Cl (Step 1). This order is consistent with that from the sequential extraction of Montana soil (SRM 2710), a certified reference material [19]. For another discovery, Johnston and Barnard (1979) [24] found that As and phosphorous (P) react similarly when treated by solutions containing sulfate, fluoride, bicarbonate, hydroxide and hydrogen ions (the same solutions as those in our test), and demonstrated the same order between As and P extractions for test soils (western New York soils): NaOH ≫ H_2_SO_4_ > NH_4_F > NH_4_Cl [19]. However, the order of As concentration extracted by NH_4_F and H_2_SO_4_ found in our study does not fit with that in the study of Johnston and Barnard [24]. For this issue, we can suggest the following reasons related to characteristics of As bound to Ca (H_2_SO_4_-extractable). Even though As and P are chemically very similar elements, As bound to Ca is far more soluble than P bound to Ca, so it may not be always accurate to apply the same sequential extraction protocol for both As and P as Johnston and Barnard’s study. Also, As bound to Ca is much more unstable than As bound to Al or Fe, so can be more soluble at pH ≤ 10. Consequently, As bound to Ca may already dissolve in one of easily soluble (pH 7) or NH_4_F extraction (pH 8) due to redistribution phenomena, and it probably also arises from As bound to Fe oxides, which is dissolved by sodium citrate + NaHCO_3_ + Na_2_S_2_O_4_·2H_2_O treatment [19]. For those reasons, Herrewghe *et al.* have been replaced the acid “H_2_SO_4_” extraction step by an oxidizing extraction using 8.8 mol/L H_2_O_2_ [19].

### 3.4. Leaching of As and Heavy Metals from the Mine Tailings

Leaching tests (TCLP and KSLT) of the mine tailings were performed to indirectly evaluate release and mobility of contaminants to the surrounding environment by normal rain (approx. pH 5.6) or acidic rain (under pH 5.6) [15,20]. According to TCLP, the pH of the solution produced by shaking the mine tailings with ultra-pure water was 7.21 and that of the solution acidified with HCl was 1.14, thus fluid #1 (pH 4.93 ± 0.05) was selected for the leaching test.

Table 6 shows leaching concentrations of As and heavy metals by TCLP and KSLT, compared with their criteria. Leaching concentrations (mg/L) of As and heavy metals were much lower than the criteria [e.g., As (mg/L): 0.43 for TCLP and 0.24 for KSLT (cf. criteria (mg/L): 5.0 for TCLP and 1.5 for KSLT), Pb (mg/L): 0.15 for TCLP and 0.10 for KSLT (cf. criteria (mg/L): 5.0 for TCLP and 3.0 for KSLT), Cr(VI) (mg/L): 0.42 for TCLP and 0.36 for KSLT (cf. criteria (mg/L): 5.0 for TCLP and 1.5 for KSLT)]. Especially, the leaching levels of As were in the range of 0.014–0.026%, presenting only meager proportions for the total arsenic content in the mine tailings. Kim *et al.* [26] also reported a similar result on leaching of As from mine tailings. In their study, As leaching levels were in the range of 0.0017–0.37%, when mine tailings of six types were mixed with water for 1 hr at a ratio of 1:5 as mass [26]. Even though the test conditions such as leaching solution and shaking time do not correspond to those in our study, the result also satisfies that soluble As in water occupied only minor fraction of the total As in mine tailings.

In terms of only the leaching levels, current state of the mine tailings can be regarded as stable for release risk of As and heavy metals by acidic rain. Further, this sample can be classified into a non-hazardous waste group for landfill treatment. It is, however, suggested that the mine tailings need to be treated to meet Korean soil contamination criteria (see Table 4), if it is expected to return them to the environment as a part of the soil.

The leaching concentrations of As in the both leaching tests (0.43 mg/L for TCLP and 0.24 mg/L for KSLT) were lower than those of fraction 1 and 2 in the sequential extraction (0.80 mg/L for fraction 1 and 12 mg/L for fraction 2) as shown in Table 7. For this result, we consider that the pH of each extractant presumably affected most greatly the As extraction reaction. Ghosh *et al.* [27] also reported that As leaching concentrations by TCLP was much less (10 times) than those by Landfill leachate (LL), and considered that the higher leachate pH (6.8) than that of TCLP (4.9) contributed to the result since As mobilization increases with pH increase. In addition, they commented that the TCLP is focused on only acidic conditions and poorly simulates alkaline pH, low redox potential, biological activity, long retention time, and organic composition of mature landfills [27]. It is therefore concluded that leaching concentrations of As in the fraction 1 and 2 by the sequential extraction (weak alkaline pH) were higher than those by TCLP and KSLT (weak acidic pH) because more As leaches with higher pH. Though, in the comparison of As leaching by TCLP and KSLT, TCLP with lower pH than KSLT showed very slightly higher As leaching and this result is attributed to other conditions such as the extraction time and the ratio of solid and liquid rather than the pH of extractant.

**Figure 4.**
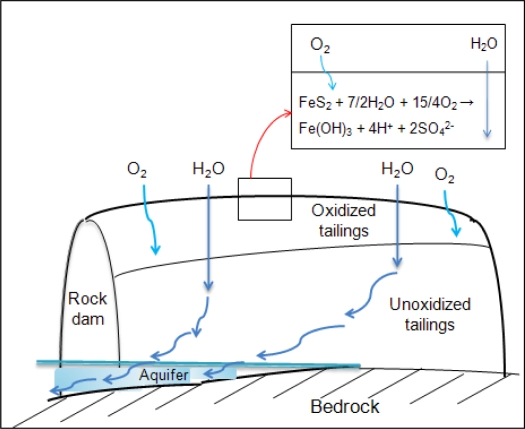
Schematic of an uncovered tailings impoundment (modified from [28]).

On the other hand, the possibility of As and heavy metals releases from the mine tailings at the natural site can be explained by comparing our study to those suggested by other studies [3,28,29]. The mine tailings used in this study were sampled from the surface of the tailings pile at the abandoned mine. Because mine tailings deposition had been ended before abandoning mining operation in 2001, the surface of uncovered mine tailings pile had been allowed to be in contact with oxygen. Those conditions resulted in the oxidation of sulfide minerals in the mine tailings, as shown in Figure 4 [28]. The oxidation of sulfide minerals which combine with As or heavy metals can also result in the dissolution of As and heavy metals by water [3]. Based on the presence of sulfide minerals, high contents of As and heavy metals, potential of oxygen contact, and high sulfate concentration in the mine tailings, the equations 6–8 related to dissolution of iron and arsenic, as an example, could have occurred in the mine tailings [3–5,29]:

(6) $${\text{FeS}}_{2}\;+\;7/2{\text{O}}_{2}\;+\;{\text{H}}_{2}\text{O}\;\to \;{\text{Fe}}^{2+}\;+\;2{\text{SO}}_{4}^{2-}\;+\;2{\text{H}}^{+}$$

(7) $${\text{Fe}}_{\left(1-\text{x}\right)}\text{S}\;+\;(2-\text{x}/2){\text{O}}_{2}\;+\;{\text{xH}}_{2}\text{O}\;\to \;(1-\text{x}){\text{Fe}}^{2+}\;+\;{\text{SO}}_{4}^{2-}\;+\;2{\text{xH}}^{+}$$

(8) $$4\text{FeAsS}\;+\;13{\text{O}}_{2}\;+\;6{\text{H}}_{2}\text{O}\;\to \;4{\text{Fe}}^{2+}\;+\;4{\text{H}}_{2}{\text{AsO}}_{4}^{-}\;+\;4{\text{SO}}_{4}^{2-}\;+\;4{\text{H}}^{+}$$

Here, ferrous ions precipitate in the form of ferric hydroxide by oxidizing or hydrating, as shown in the equation (9):

(9) $${\text{Fe}}^{2+}\;+\;1/4{\text{O}}_{2}\;+\;5/2{\text{H}}_{2}\text{O}\;\to \;\text{Fe}{\left(\text{OH}\right)}_{3}\;+\;2{\text{H}}^{+}$$

Heavy metals released by oxidation can be re-adsorbed onto the surface of ferric hydroxide, and also adsorbed heavy metals can be separated from the surface of ferric hydroxide by water [30]. The low leaching concentration in the test indicates that easily soluble fraction of As and heavy metals might be already released by the oxidation of sulfide minerals with air and water for a long period at the tailings deposition site. Based on the potential that As and heavy metals could be already released from the mine tailing, investigations on the status of contaminants in nearby lands and groundwater are needed.

## 4. Conclusions

The mine tailings were contaminated with much higher concentrations of As and heavy metals than the Korean soil contamination criteria. The measured concentrations were different depending on the test methods used. EPA method 6010 and KST for waste, digestion methods using strong acids and high temperature were more effective for total concentration analysis of As and heavy metals than KST for soil. In the mine tailings, As mostly consists of the residual fraction and the fractions extracted by alkaline extractants (NH_4_Cl and NH_4_F) were meager. In TCLP and KSLT, concentrations of As and heavy metals released from the mine tailings were far lower than their criteria, indicating a non-hazardous waste. The leaching levels of As by TCLP and KSLP were lower than those of soluble fractions by the sequential extraction because of the difference of pH among the extractants in each test. In the further study, it is necessary to investigate contamination status of nearby soil and groundwater. In addition, mine tailings treatment is needed to prevent additional oxidation of sulfide minerals in mine tailings by oxygen contact and release of As and heavy metals by water.

## Figures and Tables

**Table 1.** Comparison of US EPA method 6010 and Korean Standard Test (KST) for soil and waste.

	**EPA method 6010**	**KST for soil**	**KST for waste**
Definition and purpose	To determine concentrations of trace elements, including metals, in groundwater, soils, sludges, sediments and other solid wastes	To determine if the soil is contaminated by either inorganic or organic environmental contaminants over the regulation level by Korean soil preservation act	To determine if a waste is hazardous over the regulation level by Korean waste management act and to determine concentrations of contaminants in a waste
Target samples	Sediment, sludge, and soil	Soil	Wastes including low content of organic matter and metallic oxide, hydroxide, and sulfide
Target elements	As, Ag, Al, Ba, Be, Ca, Co, Cr, Cu, Fe, K, Mg, Mn, Mo, Na, Ni, Pb, Se, Zn, Tl, V	Cd, Cu, Pb, As	As, Pb, Cd, Cu, Cr
Sample amount	2 g	10 g	Unspecified
Reagents	1:1 HNO_3_, 30% H_2_O_2_, and concentrated HCl	0.1 N HCl (for Cd, Cu, andPb) 1 N HCl (for As)	HNO_3_ and HCl (1 + 1)
Reaction	Heating (under boiling point)	Shaking (100 rpm for 1 hr at 30 °C)	Heating (under boiling point)
Total reaction time	About 3.5 hr	1 hr	About 2 hr
Final solution volume (with water addition)	100 mL	50 mL	100 mL

**Table 2.** Comparison of Toxicity Characteristic Leaching Procedure (TCLP; EPA method 1311) and Korean Standard Leaching Test (KSLT) [16,20,21].

	**TCLP**	**KSLT**
Definition	An analysis method to determine the mobility of both organic and inorganic analytes present in liquid, solid, and multiphasic wastes	A analysis method to predict potential leaching level of environmental contaminants from industrial wastes after landfill
Purpose	To determine if a waste may meet the definition of EP (Extraction Procedure) Toxicity, that is, carrying a hazardous waste code (40CFR Part 261) under Resource Conservation and Recovery Act (RCRA)	To determine if a waste is specified over the regulation level of Korean waste management act or which landfill method is proper for a waste
Target solid materials	Materials are solid waste if they are *abandoned* by being: (1) Disposed of; or (2) Burned or incinerated; or (3) Accumulated, stored, or treated (but not recycled) before or in lieu of being abandoned by being disposed of, burned, or incinerated	Slag, dust, sand blast, waste refractory, incineration waste residue, solidified/stabilized waste, waste catalyst, waste absorbent/adsorbent, wastewater sludge, *etc.*
Sample treatment	Sieving into 9.5 mm	Sieving into 5.0–5.5 mm
Extraction device	Rotary extraction device (30 rpm)	Horizontal back-and-forth shaker (200 rpm)
Extraction time	18 hr	6 hr
pH of extractant	Fluid #1: pH 4.93 ± 0.05 Fluid #2: pH 2.88 ± 0.05	pH 5.8–6.3 adding HCl to distilled water
Sample (g): Extractant (mL)	1:20	1:10
Separation of solid and liquid	0.6–0.8 *μ*m-membrane filter or centrifuge	1 μm-membrane filter or centrifuge

**Table 3.** Physical and chemical properties of the mine tailings.

**Property**		**Unit**	**Value**
pH			7.5 ± 0.14
Water content		%	9.6 ± 0.42
Loss on ignition (LOI)		%	9.3 ± 0.33
Organic carbon content		%	3.9 ± 0.15
Anions	F^−^	mg/L	0.11
	Cl^−^	mg/L	20
	NO_2_^−^	mg/L	ND
	NO_3_^−^	mg/L	1.9
	Br^−^	mg/L	0.71
	PO_4_^2−^	mg/L	9.6
	SO_4_^2−^	mg/L	224

ND = Not Detected

**Table 4.** Total concentrations (mg/L) of arsenic (As) and heavy metals in the mine tailings compared to Korea soil contamination criteria (both acting and warning criteria).

**Metal (mg/kg)**	**Standard method**			**Korean soil contamination criteria**			
				**Acting**		**Warning**	

	EPA method 6010	KST for soil	KST for waste	A area	B area	A area	B area
As	67,336 ± 104	3,068 ± 22	66,155 ± 710	15	50	6	20
Fe	137,180 ± 756			NE	NE	NE	NE
Cu	764 ± 0.83	233 ± 1.67	745 ± 2	125	500	50	200
Pb	3,421 ± 20	875 ± 3.1	3,572 ± 51	300	1,000	100	400
Mn	24,256 ± 31			NE	NE	NE	NE
Cr(VI)	71.7 ± 0.67		65 ± 1.35	10	30	4	12
Cd	54.4 ± 0.09	7.2 ± 0.5	56.3 ± 0.3	4	30	1.5	12
Zn	12,420 ± 4.0			NE	NE	NE	NE

A area: farmland, ranch lot, school lot, park, *etc.*

B area: factory lot, railway, highway, *etc.*

NE = Not Established.

**Table 5.** Results of arsenic (As) sequential extraction of the mine tailings, given in mg/L leachate, mg/kg tailings and % of total content, according to As chemical fractions in specific six steps.

**Step** [19]	**Extractant** [19]	**As chemical fraction** [19]	**Mineral (Formula)** [25]	**As concentration** (Average)		**Proportion** (%)
				**mg/L** leachate	**mg/kg** tailings	
1. Easily soluble	1M NH_4_Cl (pH 7)	Neutral (non-ionic) As	Arsenolite (As_2_O_3_) Claudetite (As_2_O_3_)	0.80	48	0.096
2. NH_4_F-extractable	0.5M NH_4_F (pH 8)	As bound to Al	Mansfieldite (AlAsO_4_·2H_2_O)	12	716	1.4
3. NaOH-extractable	0.1M NaOH (pH 12)	As bound to Fe (non-occluded As)	Scorodite (FeAsO4·2H_2_O) Symplesite (Fe_3_(AsO_4_)_2_·8H_2_O)	89	4,343	8.7
4. Reducible	0.5M sodium citrate + 1M NaHCO_3_ + 0.5g Na_2_S_2_O_4_·2H_2_O	As bound to Fe oxide (Occluded As)	Kalfanite (Ca_2_Fe_3_O_2_(AsO_4_)·2H_2_O)	3.1	183	0.37
5. Acid soluble	0.25M H_2_SO_4_ (pH 1)	As bound to Ca	Rauenthalite (Ca_3_(AsO_4_)2·10H_2_O) Pharmacolite (Ca(HAsO_4_)·2H_2_O)	7.3	435	0.87
6. Residual	HCl_conc_ + HNO_3conc_ +HF_conc_	As bound to silicate and sulfide minerals	Arsenopyrite (FeAsS)	440	44,023	89

**Table 6.** Leaching concentrations (mg/L) of arsenic (As) and heavy metals from the mine tailings tested by Toxicity Characteristic Leaching Procedure (TCLP) and Korean Standard Leaching Test (KSLT).

**Metal**	**Leaching level (mg/L)**		**Criteria (mg/L)**	

	TCLP (US EPA)	KSLT (Korea)	TCLP (US EPA)	KSLT (Korea)
As	0.43	0.24	5.0	1.5
Pb	0.15	0.10	5.0	3.0
Cr(VI)	0.42	0.36	5.0	1.5
Cu	0.29	0.08	NE	3.0
Cd	0.20	0.19	1.0	0.3

NE = Not Established

**Table 7.** Comparison of Arsenic (As) concentrations between Toxicity Characteristic Leaching Procedure (TCLP), Korean Standard Leaching Test (KSLT), and extractable fractions (fraction 1 and 2) in the As sequential extraction.

**Parameter**	**Unit**	**TCLP**	**KSLT**	**Sequential extraction**	
				Fraction 1	Fraction 2
pH of extractant	pH	5	6	7	8
Extraction time	hr	18	6	2	15
Solid (g):liquid (mL)		1:20	1:10	1:60	1:60
As concentration in leachant	mg/L	**0.43**	**0.24**	**0.80**	**12**
